# Epigenetics and Cancer Stem Cells: Unleashing, Hijacking, and Restricting Cellular Plasticity

**DOI:** 10.1016/j.trecan.2017.04.004

**Published:** 2017-05

**Authors:** Elanor N. Wainwright, Paola Scaffidi

**Affiliations:** 1 Cancer Epigenetics Laboratory, The Francis Crick Institute, 1 Midland Road, London NW1 1AT, UK; 2 UCL Cancer Institute, University College London, London WC1E 6DD, UK

**Keywords:** cancer, epigenetics, cancer stem cells, intratumoral heterogeneity, chromatin, plasticity

## Abstract

Epigenetic mechanisms have emerged as key players in cancer development which affect cellular states at multiple stages of the disease. During carcinogenesis, alterations in chromatin and DNA methylation resulting from genetic lesions unleash cellular plasticity and favor oncogenic cellular reprogramming. At later stages, during cancer growth and progression, additional epigenetic changes triggered by interaction with the microenvironment modulate cancer cell phenotypes and properties, and shape tumor architecture. We review here recent advances highlighting the interplay between epigenetics, genetics, and cell-to-cell signaling in cancer, with particular emphasis on mechanisms relevant for cancer stem cell formation (CSC) and function.

## Trends

Epigenetic regulators are one of the most commonly mutated classes of genes in cancer. During cancer initiation, mutated epigenetic regulators lead to oncogenic cellular reprogramming and promote the acquisition of uncontrolled self-renewal. The emergence of CSCs requires elaborate reorganization of the epigenome.

During cancer growth, epigenetic mechanisms integrate the effect of cell-intrinsic (i.e., subclonal mutations) and cell-extrinsic (i.e., signaling from the microenvironment) changes and establish intratumoral heterogeneity, either promoting or inhibiting the CSC state.

‘Loose’ epigenetic constraints in cancer cells enhance cellular plasticity and allow reversible transitions between different phenotypic states. Enhanced cellular plasticity favors cancer cell adaptability and resistance to therapy.

Modulation of epigenetic processes allows targeting of the most downstream determinants of the CSC state.

## Epigenetic Changes in Cancer Initiation and Maintenance

The molecular makeup of a cancer is the result of multiple changes occurring progressively during its lifetime. Early in the disease, alterations in key genes disrupt normal cell function and endow cells with the ability to initiate a tumor or a hematological malignancy. Subsequently, as a cancer grows, additional changes superimpose onto the initiating events and affect the biological properties of cells, either enhancing or inhibiting their malignant properties. As a result of this constant modulation of cell function, tumors comprise a remarkable collection of distinct cellular phenotypes which differentially contribute to disease progression.

**Intratumoral functional heterogeneity** (see Glossary) has, in part, a genetic basis. Sequencing studies have identified the presence of both clonal mutations, which represent early initiating events, and subclonal mutations that occur at later stages of cancer growth and only affect subsets of cancer cells [1]. While in some cancers distinct subclones coexist and collectively drive tumor growth [2], in others subclonal mutations confer a selective advantage and contribute to defining which cells will sustain the disease. Subclonal mutations can also be deleterious and counteract the effects of initiating mutations, resulting in loss of malignant properties in some cells [1].

Similarly, **epigenetic mechanisms** involving DNA methylation and chromatin play a diverse role in cancer by promoting, sustaining, enhancing, or inhibiting malignant phenotypes at various stages of the disease. Research over the past decade has revealed that both cell-intrinsic (i.e., mutations) and cell-extrinsic (i.e., environmental cues) mechanisms modulate the epigenome of cancer cells, and their combined effect determines which cells preserve the self-renewal capacity acquired during tumorigenesis. These cells, referred to as **cancer stem cells** (CSCs) or leukemic stem cells (LSCs), are those responsible for driving long-term cancer growth and disease progression [3]. CSCs/LSCs evolve over time as a consequence of genetic heterogeneity, that generates self-renewing subclones with diverse fitness, and environmental changes that modulate their phenotype [3]. In this review we discuss mechanisms and implications of the emerging role of epigenetics in the formation and function of CSCs/LSCs, focusing on how changes in DNA methylation and chromatin affect **cellular plasticity** at various stages of the disease.

## Epigenetic Mechanisms Promoting the Acquisition of Uncontrolled Self-Renewal and CSC Formation

The recent identification of driver mutations affecting a wide range of epigenetic regulators in hierarchically organized cancers provides direct evidence for the importance of epigenetic dysregulation in the formation of CSCs. These mutations are typically clonal and promote the acquisition of uncontrolled self-renewal.

### Chromatin-Related Drivers Inducing CSC Formation

Leukemias represent a paradigm of hierarchical cancers maintained by LSCs. Numerous studies have identified mutated epigenetic regulators that favor the acquisition of uncontrolled self-renewal ability and initiate the disease. A prominent example is offered by mixed lineage leukemia (MLL)-associated leukemia, which is characterized by chromosomal rearrangements involving the *KMT2A*/*MLL* gene. *KMT2A*/*MLL* encodes a histone methyltransferase that orchestrates several essential cellular processes through modification of chromatin, mainly regulating accessibility to enhancer regions [4]. Oncogenic MLL fusion proteins created by translocations induce LSC formation in acute myeloid leukemia (AML) and acute lymphoblastic leukemia (ALL) 4, 5. Importantly, MLL fusion proteins can initiate the oncogenic process both in hematopoietic stem cells and in short-lived progenitors, suggesting that they are able to reprogram committed cells and actively confer *de novo* self-renewal capacity 6, 7, 8. Notably, efficient leukemic reprogramming of myeloid progenitors by MLL chimeras requires the action of a MLL antagonist, the Polycomb group (PcG) protein BMI1, to ensure repression of tumor-suppressor genes that would otherwise counteract the effect of the oncogenic fusions 9, 10. Furthermore, the observations that different cell types respond differently to the presence of MLL fusion proteins [11], and that diverse types of leukemia can be induced by the same oncoprotein, suggest that the epigenetic landscape of the cancer cell-of-origin may influence the effect of MLL chimeric proteins 12, 13. Recurrent mutations in MLL proteins have also been identified in a variety of solid tumors 14, 15, 16, 17, pointing to MLL proteins as general cancer drivers. In addition to histone modifiers, mutated structural proteins regulating the higher-order structure of chromatin, such as cohesins, have been shown to enforce stem cell transcriptional programs and have been implicated in the emergence of LSCs [18]. Moreover, inactivating mutations disrupting the function of chromatin-remodeling complexes, which are found at high frequency in various types of cancers, have been linked to aberrant activation of stem cell-related pathways 19, 20, 21.

Probably the most compelling evidence supporting a key role of chromatin in the acquisition of uncontrolled self-renewal comes from studies in glioblastoma (GBM), a highly aggressive form of brain cancer characterized by an undifferentiated phenotype and a high frequency of CSCs. Recent sequencing efforts have identified gain-of-function mutations in genes encoding histone H3 in about one third of pediatric GBMs. The gene mainly affected is *H3F3A*, and a K27M substitution is the most common alteration 22, 23. The primary mechanism leading to oncogenesis induced by the K27M mutation is inhibition of the Polycomb repressive complex 2 (PRC2), which results in genome-wide reduction in the repressive histone H3 trimethylated lysine 27 (H3K27me3) mark [24] and re-establishment of an earlier developmental program in neural precursor cells and consequent acquisition of oncogenic self-renewal ability [25]. The identification of a histone protein as a key driver in GBM has particularly important implications because it demonstrates a direct and major role of chromatin in the emergence of CSCs. Interestingly, H3.3 mutations are only found in pediatric GBM, suggesting a different mechanism of CSC formation in adult patients. Nevertheless, chromatin dysregulation is likely to play a crucial role in adult GBM as well, considering that approximately half of adult GBMs harbor mutations in at least one chromatin modifier [26].

### DNA Methylation-Related Drivers Inducing CSC Formation

Proteins involved in the establishment and maintenance of DNA methylation have also been identified as drivers of CSC formation. The methylation status of CpG dinucleotides depends on the action of DNA methyltransferases (DNMT1, DNMT3A, and DNMT3B), which apply the methyl-group to cytosines, and methylcytosine dioxygenases (TET1 and TET2), which convert 5-methylcytosine to 5-hydroxymethylcytosine and initiate a demethylation process. DNMT3A is the DNMT most commonly affected by DNA lesions, being mutated in ∼25% of AML patients [27]. Most DNMT3A mutations appear to inhibit the enzyme activity and lead to expansion of pre-LSCs, although the exact mechanism underlying this process is not fully understood and both DNA methylation-dependent and -independent mechanisms have been described 28, 29, 30, 31, 32. Interesting, loss-of-function mutations in TET proteins, which antagonize the function of DNMTs, and mutations in IDH proteins, which indirectly affect DNA methylation patterns, also lead to the expansion of pre-LSCs, suggesting that disruption of DNA methylation via multiple mechanisms can have similar consequences [27]. The role of aberrant DNA-methylation patterns in the early stages of tumorigenesis is not limited to leukemia, and mutations in DNMTs and IDHs have been also observed in solid tumors 33, 34.

**Figure 1:**
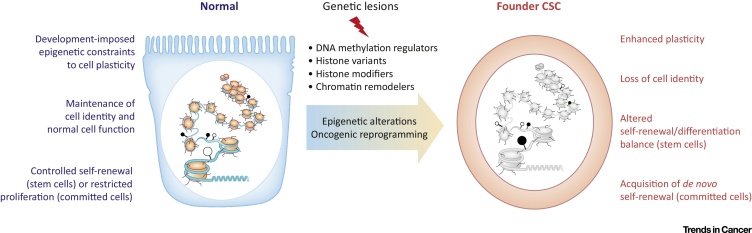
Oncogenic Reprogramming Induced by Mutated Epigenetic Regulators. Genetic alterations in chromatin-related proteins and factors involved in the establishment and maintenance of DNA methylation lead to disruption of epigenetic regulation in either adult stem cells or committed cells, and promote neoplastic transformation. The normal function of epigenetic mechanisms (left, in blue) and the consequences of epigenetic alterations induced by mutations (right, in red) are indicated. Normal or altered chromatin in the cell nucleus is depicted in color or in grey, respectively. Empty and black circles represent unmethylated and methylated CpGs, respectively. Chromatin image adapted from the webpage of the laboratory of S. Tang (www.personal.psu.edu/sxt30/projects_chromatinenzymes.html). Abbreviation: CSC, cancer stem cell.

The studies discussed above are only selected examples illustrating how mutations in epigenetic regulators crucially contribute to the formation of the founder CSC in various malignancies. As a group, epigenetic regulators are among the most commonly mutated proteins, both in individual cancer types and in pan-cancer cohorts 35, 36. Some of these genetic lesions lead to oncogenic gain of function such as those generating MLL fusions, while others damage proteins that act as tumor suppressors. Regardless of the type of mutations, a common consequence of such alterations is a global reorganization of the epigenome and consequent disruption of differentiation programs. Mutations can occur in normal stem cells, where they mainly disrupt the balance between self-renewal and differentiation, or in committed cells, where they induce a reprogramming process conferring *de novo* self-renewal capacity. In both cases, epigenetic constraints imposed during development to keep cellular plasticity under control are disrupted, and cells transform, losing their normal cellular identity (Figure 1).

## Epigenetic Mechanisms Affecting CSC Maintenance

The acquisition of uncontrolled self-renewal is only the first step in the development of a cancer. Transformed cells need to maintain their ability to self-renew while the cancer grows, a task that is evidently not trivial considering that CSCs are often only a small subset of the cancer cell population and that most cells lose self-renewal capacity over time.

### Transcriptional Intratumoral Heterogeneity

The presence of at least two functionally distinct subsets of cells (tumorigenic self-renewing CSCs, and non-tumorigenic differentiated cells) in many cancers has been the basic observation supporting the hierarchical model of cancer development over the past two decades. Recent single-cell transcriptomic studies have extended our understanding of the gene expression programs that shape tumor architecture and have confirmed that aberrant differentiation programs establish cellular hierarchies within individual cancers. In a pioneering study, colon cancers were shown to contain multiple cell types with transcriptional profiles resembling those of the cellular lineages making up the normal epithelium. Importantly, single cancer cells could recapitulate the lineage diversity of the primary tumors in transplantation assays, demonstrating that multilineage differentiation represents a key source of intratumoral transcriptional heterogeneity [37]. Similar studies in glioblastoma and oligodendroglioma have confirmed this finding, showing that brain tumors contain subpopulations of undifferentiated cells characterized by stem cell and proliferation gene signatures, and subsets of cells that have lost self-renewal potential through neural differentiation 38, 39. The approach used in these studies is powerful because it allows reconstruction of cellular hierarchies from genome-wide expression signatures in primary tumors, avoiding the caveats associated with xenograft assays. However, one limitation of these studies is their descriptive nature, which cannot assign causality to the identified gene signatures in the absence of functional validation.

**Figure 2:**
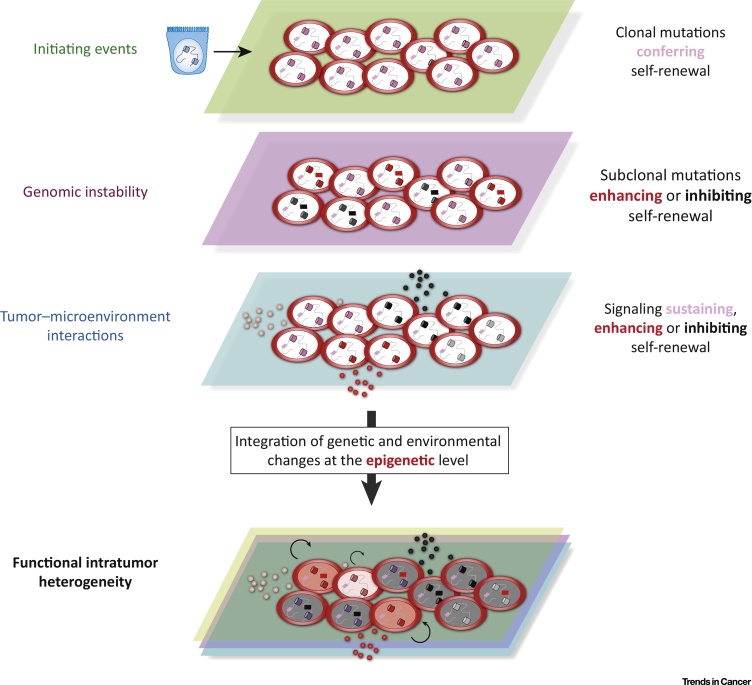
Epigenetic Mechanisms Integrate Cell-Intrinsic and Cell-Extrinsic Changes Affecting Cancer Cells and Generate Functional Intratumoral Heterogeneity. Schematic depiction of the distinct layers of alterations that affect cells during cancer development via epigenetic mechanisms. Initiating mutations (pink dash inside the nuclei) affect the cell epigenome (cylinders inside the cell nuclei) either directly, when mutations hit epigenetic regulators, or indirectly, when mutations in other drivers trigger gene expression changes mediated by chromatin remodeling and DNA methylation [100]. In either case, epigenetic reprogramming translates mutations into malignant phenotypes and promotes the acquisition of uncontrolled self-renewal. Secondary mutations occurring during tumor growth (red and black dashes) and signals from tumor microenvironment (pink, red, and black dots outside cells) induce further changes in the epigenome of the cells, either enhancing (red) or inhibiting (black) cancer cell self-renewal in a subclone- and context-dependent manner. The phenotype of each cell within a tumor is the result of all these alterations, which collectively shape the epigenome of the cell and determine which cells drive cancer growth. In the combined layers, the cell with a pink nucleus and a thin arrow represents a cell that has maintained the self-renewal ability conferred by the initiating events. Cells with red nuclei and thick arrows represent cells with enhanced self-renewal ability due to either favorable secondary mutations (cell on the right) or signaling (cell on the left). Cells with grey nuclei represent cells that have lost self-renewal ability due to either deleterious secondary mutations or signaling.

The observation that IDH-induced oligodendrogliomas contain differentiated, non-self-renewing cells underscores the diverse, and at times antithetic, role of epigenetic mechanisms in cancer development. During tumorigenesis, mutations in IDH proteins drive CSC emergence partly through alteration of DNA methylation profiles and epigenetic reprogramming of committed oligodendrocytes. However, during tumor growth, additional epigenetic changes occur and establish developmental hierarchies that restrict the proliferative potential of some cells, counteracting the effect of the initiating mutations (Figure 2).

### Epigenetic Regulators That Inhibit Cancer Cell Self-Renewal and Establish Cellular Hierarchies

What are the mechanisms that generate distinct epigenetic states within tumors and confer distinct functional properties to CSCs and differentiated cells? Epigenetic regulators belonging to two distinct groups have so far been identified: those that inhibit cancer cell self-renewal and establish differentiation hierarchies, and those that are hijacked by CSCs to avoid differentiation and sustain their phenotype (Table 1). Within the first group, the linker histone H1.0 plays an important role in restricting cancer cells long-term proliferative potential and determines functionally distinct subsets of cells within individual tumors [40]. In numerous cancer types, H1.0 levels are highly heterogeneous, with low levels in cells expressing CSC markers and high levels in differentiated cells. Functional characterization of the impact of H1.0 expression on cancer cells revealed that only cells able to stably repress H1.0 preserve a chromatin configuration compatible with self-renewal capacity. Re-expression of H1.0 in subsets of cells during tumor growth induces genome-wide silencing of oncogenic and self-renewal genes, and promotes differentiation into non-tumorigenic cells [40]. Thus, only cells expressing low H1.0 levels can maintain CSC properties. As discussed above for mutations in H3.3, the observation that an integral component of chromatin acts as an important regulator of cancer cell differentiation states highlights the important role of epigenetics in specifying tumor organization and affecting tumor maintenance. Another example supporting this concept comes from MLL-driven AML, in which high levels of the histone demethylase KDM5B revert the histone modification patterns and gene expression programs established by MLL during leukemogenesis, thereby extinguishing LCS potential [41]. Similarly, in glioma the histone methyltransferase G9a and the related global increase in H3K9me2 inhibit self-renewal of CSCs *in vitro* [42]. Although the presence of cellular hierarchies is recognized in many cancers, our knowledge of how they are established and how cancer cells lose self-renewal capacity during tumor growth is still rudimental. A comprehensive identification of the molecular players that drive cancer cell differentiation is central to understanding, and possibly exploiting, the mechanisms that naturally inhibit tumor maintenance and deprive cancer cells of their malignant properties.

**Table 1:** Non-Mutated Epigenetic Regulators Affecting Cancer Cell Self-Renewal and Plasticity^a^

Protein	Cancer type	Evidence for functional role	Effect of inhibition	Preclinical evidence using small molecules	Clinical trial	Refs
Proteins inhibiting CSC self-renewal						
H1.0	Breast cancer, Glioma and glioblastoma, Melanoma, Kidney renal papillary cell carcinoma, Liver cancer	Differential expression in CSCs (low levels) and differentiated cells (high levels); KD and OE studies with human cancer cells in graft models; Patient stratification and prognostic value	Increased frequency of self-renewing tumor cells	N/A	N/A	[40]
KDM5B/JARID1B	Acute myeloid leukemia	Differential H3K4me3 levels in LSCs (high levels) and differentiated cells (low levels); OE and KD studies *in vitro* and *in vivo* with mouse and human cells in graft models	Enhanced LSC self-renewal	N/A	N/A	[41]
EHMT2/G9a	Glioma	Loss of H3K9me2 in CSCs; OE and pharmacological inhibition studies *in vitro*	Enhanced CSC self-renewal	N/A	N/A	[42]
Proteins sustaining CSC self-renewal						
EZH2	Glioblastoma, Breast cancer, Liver cancer, Non-small cell lung cancer, Ovarian cancer	Upregulation in CSCs; OE, KD, and pharmacological inhibition studies *in vitro* and in graft models	Impaired CSC self-renewal and delayed tumor initiation	DNZep and GSK126: Increased apoptosis of NSCLC cells and sensitivity to topoisomerase ll inhibitors *in vitro* and *in vivo;* Regression of *ARID1A* mutated ovarian tumor mouse graft models	Tazemetostat (EPZ-6438): Phase I and II studies in solid tumors (NCT02875548, NCT02601950, NCT02860286, NCT02601937)	43, 44, 45, 46, 47, 48, 49, 50
Non-canonical PRC1.1	Acute myeloid leukemia	Upregulation of PRC1.1 complex components in human LSCs; KD studies in human LSCs *in vitro* and in graft models	Reduced cell proliferation	N/A	N/A	[51]
BMI1	Glioblastoma, Acute myeloid leukemia, Colon cancer	KD studies *in vitro* and in graft models and pharmacological inhibition studies	Impaired self-renewaland differentiation	PTC-209: Impaired tumor growth in colorectal cancer	PTC596: Phase I study in advanced solid tumors (NCT02404480)	9, 10, 52, 102
DOT1L	MLL-driven leukemia	Increased H3K79me in LSCs KO studies with MLL-AF9 transformed mouse cells; KD studies in graft models and pharmacological inhibition studies	Apoptosis, cell cycle arrest and differentiation	EPZ004777: Apoptosis of MLL-driven leukemia cells and increased survival in graft models; SGC0946: Synergy with BRD4 inhibition in reducing cancer growth	EPZ-5676: Phase I studies in AML and ALL (NCT02141828, NCT01684150)	57, 103, 104
MLL5	Glioblastoma	KD and OE studies with human primary GBM cultures *in vitro* and in graft models	Differentiation	N/A	N/A	[54]
KDM2A/MLL	Glioblastoma	Upregulation in GBM CSCs; KD studies in GMB cells *in vitro*	Decreased proliferation	N/A	N/A	[55]
KDM1A/LSD1	Small cell lung cancer, Acute myeloid leukemia	Upregulation in lung cancer cell lines and LSCs; Pharmacological inhibition studies *in vitro* and in graft models	Growth inhibition, differentiation, apoptosis	Tranylcypromine analogs: Inhibition of tumor growth in graft models	GSK2879552: Phase I studies in AML and SCLC (NCT02177812, NCT02034123)	56, 64
PRMT5	Chronic myeloid leukemia, Acute myeloid leukemia, Glioblastoma, Lymphoma	Upregulated in GBM CSCs; KD and pharmacological inhibition studies *in vitro* and in graft models	Impaired self-renewal, growth inhibition, apoptosis	PJ-68: Inhibition of engraftment of human CML cells in mice	GSK3326595: Phase I study in solid tumors and non-Hodgkin's lymphoma (NCT02783300)	60, 61, 62, 63
SMARCA4/BRG1	Acute myeloid leukemia	KD studies with mouse MLL-AF9/NrasG12D AML cells *in vitro* and in graft models	Growth inhibition, inhibition of disease progression, increased survival	N/A	N/A	[65]
BRD4	MLL-driven leukemia, Breast cancer, Prostate cancer, Medulloblastoma, Lung adenocarcinoma	KD studies and pharmacological inhibition studies using mouse and human cells *in vitro* and in graft models	Impaired self-renewal, induction of cell cycle arrest and apoptosis	GSK1210151A (I-BET151) and JQ1: Inhibition of tumor growth and increased survival in graft models	GSK525762: Phase I and II studies in solid tumors or hematologic malignancies (NCT01943851, NCT02964507, NCT01587703); OTX015: Phase I studies in solid tumors or hematologic malignancies (NCT01713582, NCT02259114, NCT02698176, NCT02698189); CPI-0610: Phase I and II studies in peripheral nerve sheath tumors or hematologic malignancies (NCT02986919, NCT01949883, NCT02158858, NCT02157636)	66, 67, 68, 69, 70, 71, 72, 77, 78
KDM5B/ARID1B	Melanoma	Dynamic expression within melanoma cell populations; KD studies *in vitro* and in graft models	Impaired tumor maintenance	N/A	N/A	[96]
Proteins mediating drug tolerance						
KDM5A/JARID1A	Non-small cell lung cancer	Upregulated in drug-tolerant cells *in vitro;* OE and KD studies *in vitro*	Reduced drug tolerance	N/A	N/A	[97]
KDM6A/B	Glioblastoma	KO studies in human GBM CSCs *in vitro*	Growth inhibition of drug-tolerant cells, reduced emergence of resistant cells	GSKJ4: Growth inhibition of cells tolerant to other drugs	N/A	[98]

a KD, knockdown; KO, knockout, N/A, not available; OE, overexpression. The registered clinical trial identifier (NCT) is indicated (www.clinicaltrials.gov).

### Epigenetic Regulators That Sustain Cancer Cell Self-Renewal

Although many cancer cells succumb to differentiation during tumor growth, CSCs evade this process and preserve their self-renewal capacity acquired during transformation. Not surprisingly, considering the importance of epigenetic regulators in normal stem cell maintenance, many chromatin-related proteins and DNA-methylating enzymes are essential to maintaining the CSC state. Remarkably, most of the proteins implicated in CSC maintenance are typically not mutated but are co-opted in their wild-type state by CSCs to avoid differentiation and sustain their malignant properties. Prominent examples of hijacked proteins are Polycomb complex proteins. The PRC2 catalytic subunit EZH2 has a tumor-promoting role in many malignancies, and inhibition of its activity or genetically induced loss of the protein strongly impairs tumor growth 43, 44, 45, 46, 47, 48, 49, 50. Several molecular mechanisms, not always dependent on EZH2 methyltransferase activity, have been shown to underlie the role of EZH2 in cancer, including repression of tumor suppressors 43, 44, 45, 46, 47, 48, 49, 50, activation of oncogenic NOTCH signaling [47], stabilization of β-catenin [48], and inhibition of DNA damage repair and consequent induction of secondary mutations [50]. Regardless of the specific mechanism, the commonality in all these cases is that cells become dependent on EZH2 to preserve their self-renewal potential, as a consequence of the cellular changes induced by transformation and other alterations occurring during tumor growth. Other PcG proteins such as a non-canonical PRC1.1 complex in AML [51] and BMI1 in glioma [52] exert similar functions. Histone modifiers in general have often been reported as positive regulators of CSC self-renewal (Table 1) 53, 54, 55, 56, 57, 58, 59, 60, 61, 62, 63, 64. Furthermore, the chromatin-remodeling complex SWI/SNF sustains high levels of c-MYC by regulating enhancer function and is required for maintenance of self-renewing LSCs in MLL-driven leukemia [65]. Interestingly, in many cases, functional dependency on chromatin regulators is observed in neoplasms initiated by mutations targeting other epigenetic regulators, highlighting the complex role of epigenetics in cancer and the existence of synthetic lethality interactions that could be exploited for therapeutic purposes 44, 45, 46, 53, 57, 64.

### Epigenetic Modulation of the CSC State

A key feature of epigenetic mechanisms is their inherent reversibility. Thus, the dependence of CSCs on epigenetic regulators offers an opportunity to target their self-renewal capacity. The chromatin ‘reader’ BRD4 best illustrates this concept [66]. BRD4 belongs to the BET family of chromatin readers which bind to acetylated promoters and enhancers and sustain transcription of the corresponding genes. Many key oncogenes, the most notable example being c-MYC, are among BRD4 targets, and pharmacological inhibition of BRD4 binding to their regulatory regions strongly reduces their expression levels and inhibits the growth of various cancer types 67, 68, 69. Pioneering studies in MLL-driven ALL demonstrated that BRD4 inhibitors (BETis) effectively target CSCs 67, 68, a finding confirmed in other cancer types 70, 71. The striking preclinical results obtained with BETis, reinforced by early clinical evidence [72], highlight the potential of targeting epigenetic mechanisms, showing how c-MYC, a classical untargetable oncoprotein key to many CSCs, can effectively be eliminated through interference with its upstream regulator. The case of BRD4 is remarkable in that it appears to affect the maintenance of a wide range of cancers. However, several successful preclinical studies focused on inhibition of other chromatin-related proteins in specific cancer types have confirmed the therapeutic value of targeting epigenetic regulators and have laid the foundation for ongoing clinical trials 57, 60, 73 (Table 1).

As with any therapeutic strategy, epigenetic modulation of CSCs faces challenges. Early concerns regarding targeting wild-type proteins that exert pleiotropic functions in normal cells have been mitigated by the observation that many tested inhibitors are not associated with major toxicity, suggesting that cancer cells exhibit a specific epigenetic vulnerability [74]. However, there is evidence that BETis, for example, may have deleterious effects 75, 76 and treatment regimens will need to be carefully adjusted to avoid long-term side-effects. Furthermore, while the development of resistance is theoretically less likely when targeting downstream epigenetic regulators compared to treatments interfering with upstream cellular components – extracellular or cytoplasmic proteins that can be easily bypassed using parallel pathways – resistance also can emerge when targeting nuclear components. At least two distinct mechanisms of acquired resistance to BETis have been identified in AML and breast cancer 77, 78, although characterization of the resistance mechanisms has suggested combination strategies that may enhance the clinical utility of BRD4 inhibition. Finally, despite successful preclinical studies, consistent efficacy of epigenetic drugs, such as HDAC inhibitors and DNA methylation inhibitors, has not always been observed in patients, especially in solid tumors 79, 80.

## Cell-Extrinsic Mechanisms Affecting CSC Function and Maintenance through Epigenetics

A key question related to epigenetic regulation of the CSC state is: what determines epigenetic heterogeneity within tumors? For example, why do some cells express high levels of histone H1.0 and consequently differentiate, and others instead maintain low H1.0 levels and thus self-renewal capacity [40]? Similarly, why are *HOX* oncogenes methylated in their promoter regions in particular AML cells and not in others [81]? Various pieces of evidence indicate that genetic differences alone cannot account for the observed degree of diversity, and that cell-extrinsic mechanisms play an important role. Tumors comprise a diverse ecosystem of cancer cells surrounded by vasculature, cancer-associated fibroblasts, and infiltrating immune cells. This microcosm provides numerous cell-to-cell signals that modulate gene expression programs in cancer cells independently of their genetic background and, as a consequence, affect the number, phenotype, and function of CSCs [82].

### Interplay between Extracellular Signaling and Epigenetics in Generating Intratumoral Functional Heterogeneity

**Figure 3:**
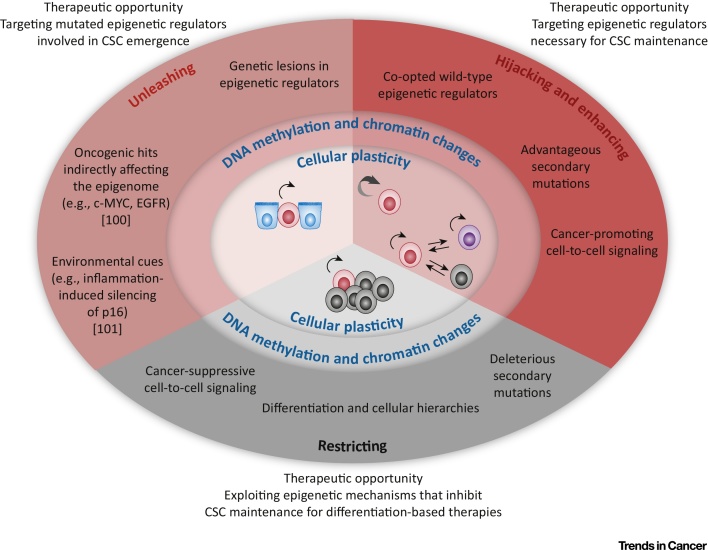
Key Figure: The Complex Role of Epigenetic Mechanisms in Cancer

Variations in oxygen and nutrient concentrations are a likely source of phenotypic variation within tumors, and histone modifiers have been reported to act as sensors of hypoxia [83] or are linked to metabolism [84]. Similarly, DNA methylation patterns are affected by changes in the environment [85]. Thus, cells exposed to distinct conditions in their local environment may respond by modifying their epigenome. Furthermore, classic cell-to-cell signaling pathways, such as WNT, TGF-β, SHH, and NOTCH, which tightly regulate self-renewal and differentiation during embryonic development and adult tissue homeostasis, affect CSC self-renewal either positively or negatively [86]. In physiological conditions, the heritable switch from division to differentiation induced or prevented by these pathways is determined by changes in chromatin and DNA methylation [87]. Although the downstream effectors of aberrant developmental pathways in cancer are largely unknown, it stands to reason that epigenetic factors are likely to be crucial mediators that translate extracellular signaling into differential phenotypes within cancers. In line with this view, functional interactions between TGF-β and two histone modifiers, PRMT5 [88] and G9a [89], modulate cancer cell phenotypes in various types of carcinoma through changes in histone methylation. Moreover, the transcriptional regulator CDK9 has been found to act as a downstream effector of the NOTCH pathway to sustain GBM stem cells [90]. In addition, cAMP, a second messenger involved in transduction of various extracellular signals, promotes breast CSC differentiation via the histone demethylase PHF2 [91]. Because chromatin-based mechanisms represent the last level of signal transduction cascades, and several ‘epigenetic’ drugs have already been developed, finding novel mechanisms where chromatin-related factors mediate CSC-sustaining signaling pathway activity could have important therapeutic implications (Figure 3, Key Figure). As an additional layer of complexity, distinct epigenetic states can in turn determine the response to microenvironment signaling, as exemplified by breast and glioma CSCs in which the tumor-suppressive or tumor-promoting effect of paracrine TGF-β signaling is determined by the DNA methylation status of SMAD target genes 92, 93. Thus, the interplay between signaling and the epigenome has emerged as a crucial force that shapes tumor architecture, and future characterization of these complex interactions will certainly provide invaluable insights into CSC biology (Figure 2).

### Reversible Epigenetic States and Cancer Cell Plasticity

A major difference between normal cellular hierarchies and cancer is that, although cell fate decisions triggered by environmental cues are generally stable and heritable in normal cells, cancer cells maintain an intrinsic plasticity that allows them to easily change their phenotype in response to new signals and possibly switch between cellular states. Evidence exists in some cancers that differentiated cells can reacquire self-renewal ability and revert to a CSC state (Figure 3). This occurs, for example, in basal carcinomas of the breast, in which TGF-β stimulation induces conversion of non-tumorigenic CD44^lo^ cells to CD44^hi^ CSCs. Importantly, this cellular plasticity appears to be dependent on the chromatin status of the *ZEB1* promoter because a poised, bivalent configuration allows reversion to CD44^hi^ CSCs upon stimulation, while the presence of repressive marks renders CD44^lo^ cells insensitive to TGF-β [94]. Another example highlighting how chromatin states can influence cancer cell plasticity comes from melanoma, a type of cancer that does not appear to develop in a hierarchical manner [95]. Even in the absence of ‘classical' CSCs, Roesch *et al.* identified a subpopulation of cells that is required for continuous tumor growth and is marked by the expression of the histone demethylase JARID1B. However, instead of being a stable subpopulation, JARID1B^+^ cells are a dynamic subset whose composition changes over time as cells gain and lose JARID1B expression, and transiently acquire stemness properties depending on the tumor context [96]. These observations support a model of transient stemness, sustained by dynamic epigenetic states, in which at any given time a self-renewing but changing subpopulation of cells exists among the bulk of tumor cells. An important implication of these findings, which can be extended to classical hierarchically organized tumors, is that the local tumor context appears to be key in determining, through epigenetics, which cells within a tumor can act as tumor-maintaining CSCs, arguing against a deterministic model of CSC identity.

### Adaptive Chromatin Remodeling and Resistance to Therapy

Despite significant progress in the development of effective therapies against numerous cancer types, therapeutic resistance is still relatively common. CSCs are the likely source of resistant cells responsible for disease relapse because cells deprived of self-renewing potential are unable to reconstitute the cancer even if they survive treatment. While drug resistance primarily has a genetic basis, chromatin-related mechanisms have emerged as additional players in this context that are exploited by cancer cells to enhance their plasticity and adaptability (Figure 3). A paradigm of epigenetically driven resistance has recently been provided by Sharma *et al.* who reported the existence of a reversible drug-tolerant state in non-small cell lung cancer (NSCLC)-derived cell lines, and which can survive exposure to lethal concentrations of EGFR tyrosine kinase inhibitors. This drug-tolerant state does not involve drug efflux and is instead associated with an altered chromatin state and requires the histone demethylase KDM5A/JARID1A and IGF-1R signaling [97]. Similarly, upon treatment with targeted kinase inhibitors, GBM CSCs can reversibly transition to a slow-cycling persistent state in which primitive developmental programs are upregulated. The parallels with NSCLC extend to the dependence on a specific signaling pathway, NOTCH, and a histone demethylase, KDM6A/B [98]. In both cases it is hypothesized that this reversible chromatin state allows cancer cells to survive the initial lethal stress before further, more permanent, resistance mechanisms can evolve. As an additional example, in *NOTCH1*-driven T cell ALL, clinical trials using γ-secretase inhibitors (GSI) have shown limited efficacy owing to the presence of a reversible subpopulation of GSI-tolerant cells characterized by BRD4-dependent transcriptional programs. Notably, combined treatment of patient-derived xenografts with NOTCH and BRD4 inhibitors showed greater efficacy than individual treatments [99]. Overall, these studies reveal that chromatin-related mechanisms frequently characterize therapeutic resistant cancer cell subpopulations, and support the use of epigenetic-targeting drugs in combination therapies as a means to overcome drug resistance (Figure 3).

## Concluding Remarks

Epigenetic alterations affecting chromatin and DNA methylation patterns are universal features of cancer. Historically, it has been difficult to distinguish whether these changes play a functional role in the disease or are a bystander phenomenon that merely reflects alterations in cell behavior. However, studies over the past 5–10 years have crystallized the importance of epigenetic mechanisms at various stages of cancer development and have uncovered unprecedented therapeutic opportunities (Figure 3). The realization that genes involved in epigenetic regulation are among the most commonly mutated gene families has profound implications for understanding the mechanistic basis of carcinogenesis, and these studies have revealed that interference with differentiation programs is a major mechanism leading to CSC emergence. Complementary to cancer genomics studies, the identification of epigenetic regulators hijacked by CSCs to sustain their phenotypes, and the functional characterization of cell-intrinsic mechanisms that establish and maintain cellular hierarchies within cancers, have demonstrated the crucial role of epigenetics in tumor maintenance and progression. Given the reversible nature of epigenetic mechanisms, these findings have enormous therapeutic potential (Figure 3). The impressive preclinical results obtained with BET inhibitors provide a paradigm illustrating the power of epigenetic therapy. It is highly likely that additional proteins may exert similarly important, and possibly widespread, roles in tumor maintenance. For instance, very little is known about the downstream effectors that mediate CSC-sustaining cell-to-cell signaling. The challenge in identifying such proteins is the absence of mutations and, often, of transcriptional alterations, which make them ‘invisible’ to unbiased genome-wide analysis. Functional screening leveraging novel technologies such as CRISPR/Cas9 genome editing may aid the identification of novel therapeutic targets, especially if coupled with CSC-relevant assays.

An alternative strategy to target CSCs, which has been explored only in part, is induction of their differentiation by enhancing the natural process that occurs *in vivo*. The presence of cellular hierarchies in cancer clearly indicates that mechanisms inhibiting cell self-renewal ability exist, and can efficiently deprive cells of their malignant properties. Differentiation therapies that ‘exaggerate’ such mechanisms and restrict cellular plasticity may prove useful to exhaust CSCs and thus halt tumor maintenance, and also to impair cancer cell adaptability (Figure 3). A prerequisite to achieve this is a comprehensive understanding of the driving forces that shape tumor architecture, and further research in this direction is essential (see Outstanding Questions).

Considering the diverse role of epigenetics in cancer, and the possible interference with normal homeostasis, epigenetic modulation of CSCs clearly still faces many challenges, but at the same time offers unprecedented opportunities to hit the beating heart of the disease.

Outstanding Questions What is the role of cellular hierarchies in cancer? Do differentiated cells help CSCs in any way, possibly through paracrine signaling – or is differentiation simply one of many anticancer safeguard mechanisms that cells must evade to establish a ‘successful’ cancer? If the latter, can epigenetic mechanisms naturally driving loss of self-renewal be exploited for therapeutic purposes?

What are the upstream mechanisms that establish epigenetic heterogeneity within individual tumors? What determines whether the different epigenetic states are stable or whether a dynamic equilibrium between self-renewing and non-self-renewing (or drug-sensitive and drug-tolerant) cells is established? Can cancer cell plasticity be modulated to reduce resistance to treatment? How prevalent are epigenetic mechanisms in the development of therapeutic resistance?

Why do cancer cells, including CSCs, show specific sensitivity to epigenetic drugs that target wild-type proteins expressed in normal cells as well? Could epigenetic drugs be generally useful as agents to be administered in combination therapies?

## Glossary

**Cancer stem cells (CSCs)**: cancer cells endowed with unlimited self-renewal potential that are responsible for tumor maintenance. Operative definition: cells that can propagate the disease when transplanted into immunocompromised mice, recapitulating the cellular heterogeneity observed in the primary tumor.
**Cellular Plasticity**: the ability of cells to transition between different phenotypic states.
**Epigenetic mechanisms**: molecular processes affecting cell behavior via changes in gene expression that do not involve genetic alterations. Epigenetic mechanisms are primarily mediated by changes in chromatin structure and DNA methylation patterns which render genes differentially competent for transcription.
**Intratumoral functional heterogeneity**: the presence of two or more subpopulations of cancer cells with distinct biological properties within the same tumor.

## References

[1] Burrell R.A. (2013). The causes and consequences of genetic heterogeneity in cancer evolution. Nature.

[2] Sottoriva A. (2015). A big bang model of human colorectal tumor growth. Nat. Genet..

[3] Kreso A., Dick J.E. (2014). Evolution of the cancer stem cell model. Cell Stem Cell.

[4] Shilatifard A. (2012). The COMPASS family of histone H3K4 methylases: mechanisms of regulation in development and disease pathogenesis. Annu. Rev. Biochem..

[5] Sanjuan-Pla A. (2015). Revisiting the biology of infant t(4;11)/MLL-AF4^+^ B-cell acute lymphoblastic leukemia. Blood.

[6] Cozzio A. (2003). Similar MLL-associated leukemias arising from self-renewing stem cells and short-lived myeloid progenitors. Genes Dev..

[7] Somervaille T.C. (2009). Hierarchical maintenance of MLL myeloid leukemia stem cells employs a transcriptional program shared with embryonic rather than adult stem cells. Cell Stem Cell.

[8] Krivtsov A.V. (2006). Transformation from committed progenitor to leukaemia stem cell initiated by MLL-AF9. Nature.

[9] Yuan J. (2011). Bmi1 is essential for leukemic reprogramming of myeloid progenitor cells. Leukemia.

[10] Smith L.L. (2011). Functional crosstalk between Bmi1 and MLL/Hoxa9 axis in establishment of normal hematopoietic and leukemic stem cells. Cell Stem Cell.

[11] Montes R. (2011). Enforced expression of MLL-AF4 fusion in cord blood CD34+ cells enhances the hematopoietic repopulating cell function and clonogenic potential but is not sufficient to initiate leukemia. Blood.

[12] Chen W. (2006). A murine Mll-AF4 knock-in model results in lymphoid and myeloid deregulation and hematologic malignancy. Blood.

[13] Krivtsov A.V. (2008). H3K79 methylation profiles define murine and human MLL-AF4 leukemias. Cancer Cell.

[14] Peifer M. (2012). Integrative genome analyses identify key somatic driver mutations of small-cell lung cancer. Nat. Genet..

[15] Parsons D.W. (2011). The genetic landscape of the childhood cancer medulloblastoma. Science.

[16] Fujimoto A. (2012). Whole-genome sequencing of liver cancers identifies etiological influences on mutation patterns and recurrent mutations in chromatin regulators. Nat. Genet..

[17] Gui Y. (2011). Frequent mutations of chromatin remodeling genes in transitional cell carcinoma of the bladder. Nat. Genet..

[18] Mazumdar C. (2015). Leukemia-associated cohesin mutants dominantly enforce stem cell programs and impair human hematopoietic progenitor differentiation. Cell Stem Cell.

[19] Jagani Z. (2010). Loss of the tumor suppressor Snf5 leads to aberrant activation of the Hedgehog–Gli pathway. Nat. Med..

[20] Wang X. (2011). TCR-dependent transformation of mature memory phenotype T cells in mice. J. Clin. Invest..

[21] Wilson B.G., Roberts C.W. (2011). SWI/SNF nucleosome remodellers and cancer. Nat. Rev. Cancer.

[22] Wu G. (2012). Somatic histone H3 alterations in pediatric diffuse intrinsic pontine gliomas and non-brainstem glioblastomas. Nat. Genet..

[23] Sturm D. (2012). Hotspot mutations in H3F3A and IDH1 define distinct epigenetic and biological subgroups of glioblastoma. Cancer Cell.

[24] Lewis P.W. (2013). Inhibition of PRC2 activity by a gain-of-function H3 mutation found in pediatric glioblastoma. Science.

[25] Funato K. (2014). Use of human embryonic stem cells to model pediatric gliomas with H3.3K27M histone mutation. Science.

[26] Brennan C.W. (2013). The somatic genomic landscape of glioblastoma. Cell.

[27] The Cancer Genome Atlas Research Network (2013). Genomic and epigenomic landscapes of adult *de novo* acute myeloid leukemia. N. Engl. J. Med..

[28] Lu R. (2016). Epigenetic perturbations by Arg882-mutated DNMT3A potentiate aberrant stem cell gene-expression program and acute leukemia development. Cancer Cell.

[29] Yang L. (2016). DNMT3A loss drives enhancer hypomethylation in FLT3-ITD-associated leukemias. Cancer Cell.

[30] Mayle A. (2015). Dnmt3a loss predisposes murine hematopoietic stem cells to malignant transformation. Blood.

[31] Koya J. (2016). DNMT3A R882 mutants interact with polycomb proteins to block haematopoietic stem and leukaemic cell differentiation. Nat. Commun..

[32] Russler-Germain D.A. (2014). The R882H DNMT3A mutation associated with AML dominantly inhibits wild-type DNMT3A by blocking its ability to form active tetramers. Cancer Cell.

[33] Kim M.S. (2013). Mutational analysis of DNMT3A gene in acute leukemias and common solid cancers. APMIS.

[34] Cohen A.L. (2013). IDH1 and IDH2 mutations in gliomas. Curr. Neurol. Neurosci. Rep..

[35] Lawrence M.S. (2014). Discovery and saturation analysis of cancer genes across 21 tumour types. Nature.

[36] Vogelstein B. (2013). Cancer genome landscapes. Science.

[37] Dalerba P. (2011). Single-cell dissection of transcriptional heterogeneity in human colon tumors. Nat. Biotechnol..

[38] Patel A.P. (2014). Single-cell RNA-seq highlights intratumoral heterogeneity in primary glioblastoma. Science.

[39] Tirosh I. (2016). Single-cell RNA-seq supports a developmental hierarchy in human oligodendroglioma. Nature.

[40] Torres C.M. (2016). The linker histone H1.0 generates epigenetic and functional intratumor heterogeneity. Science.

[41] Wong S.H. (2015). The H3K4-methyl epigenome regulates leukemia stem cell oncogenic potential. Cancer Cell.

[42] Tao H. (2014). Histone methyltransferase G9a and H3K9 dimethylation inhibit the self-renewal of glioma cancer stem cells. Mol. Cell. Biochem..

[43] Varambally S. (2002). The polycomb group protein EZH2 is involved in progression of prostate cancer. Nature.

[44] Kim K.H. (2015). SWI/SNF-mutant cancers depend on catalytic and non-catalytic activity of EZH2. Nat. Med..

[45] Fillmore C.M. (2015). EZH2 inhibition sensitizes BRG1 and EGFR mutant lung tumours to TopoII inhibitors. Nature.

[46] Bitler B.G. (2015). Synthetic lethality by targeting EZH2 methyltransferase activity in ARID1A-mutated cancers. Nat. Med..

[47] Gonzalez M.E. (2014). EZH2 expands breast stem cells through activation of NOTCH1 signaling. Proc. Natl. Acad. Sci. U. S. A..

[48] Zhu P. (2016). lnc-beta-Catm elicits EZH2-dependent beta-catenin stabilization and sustains liver CSC self-renewal. Nat. Struct. Mol. Biol..

[49] Suva M.L. (2009). EZH2 is essential for glioblastoma cancer stem cell maintenance. Cancer Res..

[50] Chang C.J. (2011). EZH2 promotes expansion of breast tumor initiating cells through activation of RAF1-beta-catenin signaling. Cancer Cell.

[51] van den Boom V. (2016). Non-canonical PRC1.1 targets active genes independent of H3K27me3 and is essential for leukemogenesis. Cell Rep..

[52] Gargiulo G. (2013). *In vivo* RNAi screen for BMI1 targets identifies TGF-beta/BMP-ER stress pathways as key regulators of neural- and malignant glioma-stem cell homeostasis. Cancer Cell.

[53] Rau R.E. (2016). DOT1L as a therapeutic target for the treatment of DNMT3A-mutant acute myeloid leukemia. Blood.

[54] Gallo M. (2015). MLL5 orchestrates a cancer self-renewal state by repressing the histone variant H3.3 and globally reorganizing chromatin. Cancer Cell.

[55] Gallo M. (2013). A tumorigenic MLL-homeobox network in human glioblastoma stem cells. Cancer Res..

[56] Mohammad H.P. (2015). A DNA hypomethylation signature predicts antitumor activity of LSD1 inhibitors in SCLC. Cancer Cell.

[57] Gilan O. (2016). Functional interdependence of BRD4 and DOT1L in MLL leukemia. Nat. Struct. Mol. Biol..

[58] Zhu Y. (2015). Brahma regulates the Hippo pathway activity through forming complex with Yki-Sd and regulating the transcription of Crumbs. Cell. Signal..

[59] Zhu P. (2016). LncBRM initiates YAP1 signalling activation to drive self-renewal of liver cancer stem cells. Nat. Commun..

[60] Jin Y. (2016). Targeting methyltransferase PRMT5 eliminates leukemia stem cells in chronic myelogenous leukemia. J. Clin. Invest..

[61] Banasavadi-Siddegowda Y.K. (2016). PRMT5–PTEN molecular pathway regulates senescence and self-renewal of primary glioblastoma neurosphere cells. Oncogene.

[62] Mavrakis K.J. (2016). Disordered methionine metabolism in MTAP/CDKN2A-deleted cancers leads to dependence on PRMT5. Science.

[63] Kryukov G.V. (2016). MTAP deletion confers enhanced dependency on the PRMT5 arginine methyltransferase in cancer cells. Science.

[64] Harris W.J. (2012). The histone demethylase KDM1A sustains the oncogenic potential of MLL-AF9 leukemia stem cells. Cancer Cell.

[65] Shi J. (2013). Role of SWI/SNF in acute leukemia maintenance and enhancer-mediated Myc regulation. Genes Dev..

[66] Dawson M.A. (2012). Targeting epigenetic readers in cancer. N. Engl. J. Med..

[67] Dawson M.A. (2011). Inhibition of BET recruitment to chromatin as an effective treatment for MLL-fusion leukaemia. Nature.

[68] Zuber J. (2011). RNAi screen identifies Brd4 as a therapeutic target in acute myeloid leukaemia. Nature.

[69] Lockwood W.W. (2012). Sensitivity of human lung adenocarcinoma cell lines to targeted inhibition of BET epigenetic signaling proteins. Proc. Natl. Acad. Sci. U. S. A..

[70] Venkataraman S. (2014). Inhibition of BRD4 attenuates tumor cell self-renewal and suppresses stem cell signaling in MYC driven medulloblastoma. Oncotarget.

[71] Shi J. (2014). Disrupting the interaction of BRD4 with diacetylated Twist suppresses tumorigenesis in basal-like breast cancer. Cancer Cell.

[72] Herait P.E. (2014). Abstract CT231: BET-bromodomain inhibitor OTX015 shows clinically meaningful activity at nontoxic doses: interim results of an ongoing phase I trial in hematologic malignancies. Cancer Res..

[73] Stewart C.A., Byers L.A. (2015). Altering the course of small cell lung cancer: targeting cancer stem cells via LSD1 inhibition. Cancer Cell.

[74] Dawson M.A., Kouzarides T. (2012). Cancer epigenetics: from mechanism to therapy. Cell.

[75] Bolden J.E. (2014). Inducible *in vivo* silencing of Brd4 identifies potential toxicities of sustained BET protein inhibition. Cell Rep..

[76] Fernandez P. (2014). Transformation resistance in a premature aging disorder identifies a tumor-protective function of BRD4. Cell Rep..

[77] Shu S. (2016). Response and resistance to BET bromodomain inhibitors in triple-negative breast cancer. Nature.

[78] Fong C.Y. (2015). BET inhibitor resistance emerges from leukaemia stem cells. Nature.

[79] Qiu T. (2013). Effects of treatment with histone deacetylase inhibitors in solid tumors: a review based on 30 clinical trials. Future Oncol..

[80] Nie J. (2014). Decitabine, a new star in epigenetic therapy: the clinical application and biological mechanism in solid tumors. Cancer Lett..

[81] Jung N. (2015). An LSC epigenetic signature is largely mutation independent and implicates the HOXA cluster in AML pathogenesis. Nat. Commun..

[82] Junttila M.R., de Sauvage F.J. (2013). Influence of tumour micro-environment heterogeneity on therapeutic response. Nature.

[83] Hancock R.L. (2015). Epigenetic regulation by histone demethylases in hypoxia. Epigenomics.

[84] Mentch S.J. (2015). Histone methylation dynamics and gene regulation occur through the sensing of one-carbon metabolism. Cell Metab..

[85] Feil R., Fraga M.F. (2012). Epigenetics and the environment: emerging patterns and implications. Nat. Rev. Genet..

[86] Takebe N. (2015). Targeting Notch, Hedgehog, and Wnt pathways in cancer stem cells: clinical update. Nat. Rev. Clin. Oncol..

[87] Mosimann C. (2009). Beta-catenin hits chromatin: regulation of Wnt target gene activation. Nat. Rev. Mol. Cell Biol..

[88] Chen H. (2017). A TGFbeta–PRMT5–MEP50 axis regulates cancer cell invasion through histone H3 and H4 arginine methylation coupled transcriptional activation and repression. Oncogene.

[89] Dong C. (2012). G9a interacts with Snail and is critical for Snail-mediated E-cadherin repression in human breast cancer. J. Clin. Invest..

[90] Xie Q. (2016). RBPJ maintains brain tumor-initiating cells through CDK9-mediated transcriptional elongation. J. Clin. Invest..

[91] Pattabiraman D.R. (2016). Activation of PKA leads to mesenchymal-to-epithelial transition and loss of tumor-initiating ability. Science.

[92] Bruna A. (2007). High TGFbeta-Smad activity confers poor prognosis in glioma patients and promotes cell proliferation depending on the methylation of the PDGF-B gene. Cancer Cell.

[93] Tufegdzic Vidakovic A. (2015). Context-specific 3ffects of TGF-beta/SMAD3 in cancer are modulated by the epigenome. Cell Rep..

[94] Chaffer C.L. (2013). Poised chromatin at the ZEB1 promoter enables breast cancer cell plasticity and enhances tumorigenicity. Cell.

[95] Quintana E. (2008). Efficient tumour formation by single human melanoma cells. Nature.

[96] Roesch A. (2010). A temporarily distinct subpopulation of slow-cycling melanoma cells is required for continuous tumor growth. Cell.

[97] Sharma S.V. (2010). A chromatin-mediated reversible drug-tolerant state in cancer cell subpopulations. Cell.

[98] Liau B.B. (2017). Adaptive chromatin remodeling drives glioblastoma stem cell plasticity and drug tolerance. Cell Stem Cell.

[99] Knoechel B. (2014). An epigenetic mechanism of resistance to targeted therapy in T cell acute lymphoblastic leukemia. Nat. Genet..

[100] Yao X. (2017). Epigenomic consequences of coding and noncoding driver mutations. Trends Cancer.

[101] Baylin S.B. (2012). The cancer epigenome: its origins, contributions to tumorigenesis, and translational implications. Proc. Am. Thorac. Soc..

[102] Kreso A. (2014). Self-renewal as a therapeutic target in human colorectal cancer. Nat. Med..

[103] Bernt K.M. (2011). MLL-rearranged leukemia is dependent on aberrant H3K79 methylation by DOT1L. Cancer Cell.

[104] Daigle S.R. (2011). Selective killing of mixed lineage leukemia cells by a potent small-molecule DOT1L inhibitor. Cancer Cell.

